# Cooperativity in *Escherichia coli* L-Threonine Dehydrogenase and Its Inhibition by an Antibacterial *N*-Pyridylpyrazolone Derivative

**DOI:** 10.3390/ijms262311751

**Published:** 2025-12-04

**Authors:** Ana Obaha, Nika Mikulič Vernik, Karmen Mlinar, Marcel Tušek, Milena Stojkovska Docevska, Nejc Petek, Jurij Svete, Marko Novinec

**Affiliations:** Department of Chemistry and Biochemistry, Faculty of Chemistry and Chemical Technology, University of Ljubljana, Večna pot 113, 1000 Ljubljana, Slovenia; ana.obaha@fkkt.uni-lj.si (A.O.); milena.stojkovska@fkkt.uni-lj.si (M.S.D.); nejc.petek@fkkt.uni-lj.si (N.P.); jurij.svete@fkkt.uni-lj.si (J.S.)

**Keywords:** allostery, enzyme kinetics, partial inhibition, mixed inhibition, homotetramer, antibiotics, mass photometry

## Abstract

Antibiotic resistance is an increasing concern in modern healthcare. Therefore, it is important to identify novel antimicrobial agents and new molecular targets for such compounds. Here, we describe the identification of an *N*-pyridylpyrazolone derivative, 4-(2-aminoethyl)-2-(pyridin-2-yl)-1,2-dihydro-3*H*-pyrazol-3-one dihydrochloride (compound **1**), which is effective against Gram-positive and Gram-negative bacteria and inhibits the enzymatic activity of *Escherichia coli* L-threonine dehydrogenase (TDH). To characterize its interaction with compound **1**, TDH was overexpressed in *E. coli*. The recombinant enzyme was shown to exist in dilute solution in equilibrium between dimeric and tetrameric forms, with a *K*_d_ value for the dimer/tetramer transition of 3 ± 1 nM, and to bind L-threonine cooperatively with a Hill coefficient of 1.4. Compound **1** acted as a partial mixed inhibitor of TDH with an *EC*_50_ value of 47 ± 16 µM and did not affect the equilibrium between oligomeric states. Altogether, these findings identify compound **1** as a promising starting point for the development of novel antibiotics and as a tool compound for studying the functional properties of TDH.

## 1. Introduction

Antibiotic resistance is becoming a pressing concern in modern healthcare, especially as novel targets and types of antibiotics have been scarce in recent decades. Therefore, it is important to explore novel ways of fighting bacterial infections and preventing bacterial growth in general. Many innovative strategies are being explored, often focusing on molecular targets essential for bacterial growth and survival that are absent in humans, thereby minimizing the risk of adverse effects.

L-threonine 3-dehydrogenases (EC 1.1.1.103; TDH) catalyze the dehydrogenation of L-threonine to 2-amino-3-oxobutanoate. TDH from *Escherichia coli* belongs to the family of zinc-containing medium-chain alcohol dehydrogenases [1,2] and initiates a major pathway of L-threonine degradation in *E. coli* [3]. It utilizes NAD^+^ as a cofactor, requires Zn^2+^ or other bivalent metal cations such as Mn^2+^ [4,5] for enzymatic activity, and is a homotetramer of about 140 kDa [6]. Apart from its housekeeping role in amino acid metabolism, TDH has been identified to be involved in the synthesis of quorum-sensing mediators called autoinducers in *Vibrio cholerae* and *E. coli*, which regulate population growth and biofilm formation [7,8]. Indeed, many mutations in TDH have been found to impair the growth of *E. coli* [9]. Together with the fact that functional TDH is absent in humans [10], this makes it a lucrative potential candidate for the development of antimicrobials.

**Figure 1:**
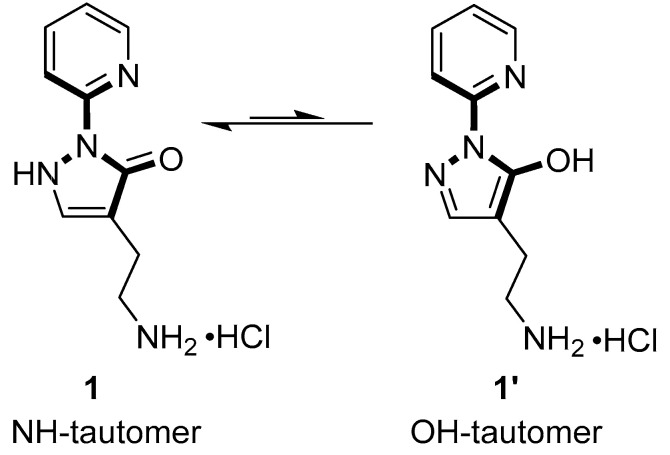
Structural formula of compound **1** and its tautomeric form **1′**. The part of the molecule resembling the structure of L-Thr is shown in bold.

In this work, we characterize the antibacterial activity of 4-(2-aminoethyl)-2-(pyridin-2-yl)-1,2-dihydro-3*H*-pyrazol-3-one dihydrochloride (compound **1**) [11,12] from our in-house compound library (https://knjiznica-spojin.fkkt.uni-lj.si/, accessed on 1 October 2022) against representative Gram-positive and Gram-negative bacteria, and we identify *E. coli* TDH as a potential target of compound **1**. Pyrazole derivatives are known for their diverse biological activities, including antibacterial properties [13,14]. Compound **1** was first described and characterized in ref. [12]. It exists in two tautomeric forms and contains a molecular fragment that resembles the structure of L-Thr (Figure 1). In continuation, we overexpress recombinant TDH, verify its basic kinetic properties and oligomeric composition, and characterize its interaction with compound **1**.

## 2. Results and Discussion

### 2.1. Identification of TDH as Target

**Figure 2:**
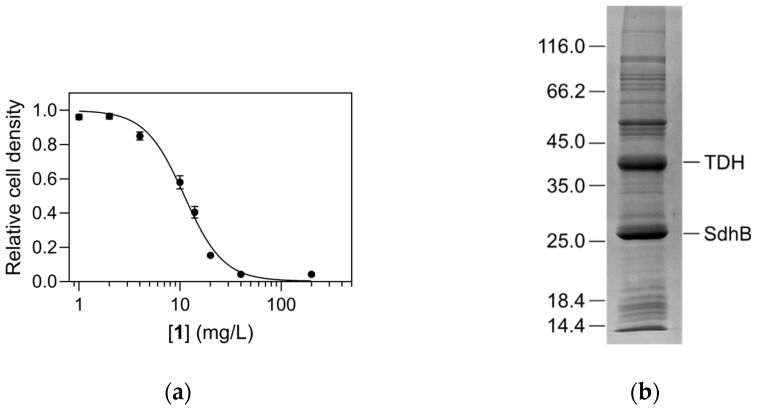
Antibacterial activity of compound **1** and its interaction with *E. coli* proteins. (**a**) Dilution antibiogram of compound **1** activity on the growth of *Escherichia coli* at 37 °C. (**b**) SDS-PAGE analysis of affinity chromatography of *E. coli* lysate on a column containing immobilized compound **1**. The denoted major bands were identified as TDH and SdhB. The positions of calibrating proteins (Unstained Protein Molecular Weight Marker, ThermoScientific, Waltham, MA, USA) are given on the left.

In a systematic screen of our in-house compound collection, compound **1** (4-(2-aminoethyl)-2-(pyridin-2-yl)-1,2-dihydro-3*H*-pyrazol-3-one dihydrochloride) exhibited weak inhibitory activity against the growth of *E. coli*, with a minimal inhibitory concentration of 40 mg/L in a dilution antibiogram (Figure 2a). In the continuation a standard Kirby–Bauer disc diffusion test was used to determine the antimicrobial properties of the compound against four different bacterial strains, including both Gram-positive and Gram-negative bacteria (Table 1). The measured zones of inhibition indicated detectable antimicrobial activity against all strains, although overall they were significantly lower than those of kanamycin, which was used as the positive control. The inhibition was strongest against *E. coli* and overall stronger against both Gram-negative strains (*E. coli* and *Pseudomonas aeruginosa*) than against the Gram-positive strains *Straphylococcus aureus* and *Bacillus thuringiensis*. Notably, compound **1** was as effective as kanamycin against *Pseudomonas aeruginosa*, which is known for its high resistance to current antibiotics [15].

Since we were interested in identifying novel macromolecular targets for antibiotic development, we aimed to identify any protein targets that specifically interact with compound **1**. This was achieved by ligand fishing, i.e., immobilization of compound **1** on an NHS-activated Sepharose carrier using its primary amino group. *E. coli* cell lysate was applied to the column, and specifically retained proteins were identified by mass spectrometry. The major bands visualized by SDS-PAGE (Figure 2b) were identified as TDH (55% sequence coverage) and subunit 2 of the succinate dehydrogenase complex, SdhB (87% sequence coverage). Given the structural similarity of compound **1** and its OH-tautomeric form **1′** [12] to L-Thr (Figure 1), the ligand could potentially act as a substrate analogue and inhibit TDH activity in a substrate-competitive manner. Therefore, the interaction with TDH was further investigated.

### 2.2. Functional Characterization of Recombinant TDH

**Figure 3:**
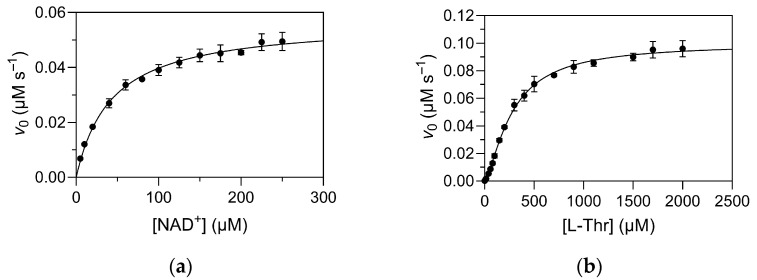
Dependence of initial reaction rates on (**a**) NAD^+^ and (**b**) L-Thr concentrations under pseudo-first order conditions. Reactions were performed at 25 °C in 50 mM Tris/HCl, pH 8.4, with a final enzyme (subunit) concentration of 10 nM. In panel (**a**), the concentration of L-Thr is fixed at 1 mM, and in panel (**b**), the concentration of NAD^+^ is fixed at 1 mM.

The kinetic parameters of TDH for the substrate L-Thr and its co-factor/co-substrate NAD^+^ have been previously reported on several occasions. Importantly, while most publications have reported the standard *K*_m_ and *V*_max_/*k*_cat_ parameters according to the Michaelis–Menten equation, some have also indicated the presence of cooperative-like effects, with a sigmoidal dependence of reaction velocity on substrate concentration [16]. Moreover, deviations from classical Michaelis–Menten kinetics have been reported in related alcohol dehydrogenases, e.g., human liver alcohol dehydrogenase γ2 [17]. Herein, we performed a detailed kinetic analysis of overexpressed TDH, which showed that the wild-type enzyme indeed exhibits cooperative binding of L-Thr, but not of NAD^+^ (Figure 3). Experiments were initially performed by varying the concentration of NAD^+^ at a fixed concentration of L-Thr (1 mM; Figure 3a), and then repeated by varying the concentration of L-Thr at a saturating concentration of NAD^+^ (1 mM; Figure 3b). The determined kinetic constants are collected in Table 2. The differences in the determined *V*_max_ values can be attributed to the relative saturations of the enzyme with the substrate used at fixed concentration in each experiment. When determining the kinetic parameters for L-Thr, the concentration of NAD^+^ was >20 × *K*_m_, which is indeed near saturation for practical purposes, whereas the concentration of L-Thr was only about 3.6 × *K*_0.5_ when determining the kinetic parameters for NAD^+^. The cooperativity factor (Hill coefficient) for the binding of L-Thr was 1.4 ± 0.1, indicating that the cooperative effect is less pronounced than in other cooperative tetramers, such as phosphofructokinase [18], hexokinase [19], and hemoglobin [20].

**Figure 4:**
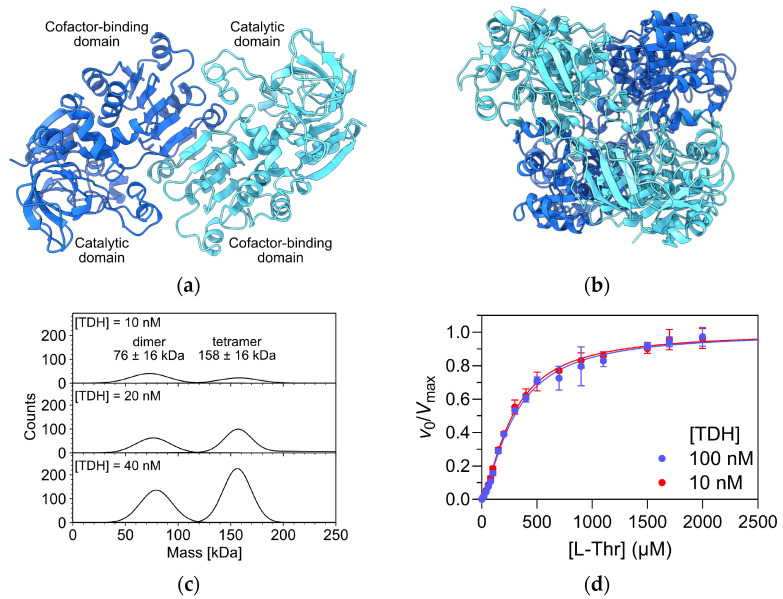
Cooperativity and oligomeric states of *E. coli* TDH. (**a**) AlphaFold 3 model of the TDH dimer. Subunits are colored dark and light blue, respectively. Domains within individual subunits are marked. (**b**) AlphaFold 3 model of the TDH tetramer. The color scheme corresponds to Figure 4a. (**c**) Mass photometry analysis of TDH at subunit concentrations between 10 and 40 nM. The analysis was performed in PBS buffer, pH 7.4, at 25 °C. (**d**) Overlay of Michaelis–Menten curves obtained at 10 nM and 100 nM subunit concentrations. The reaction conditions are the same as in Figure 2b.

The crystal structure of TDH from *E. coli* has not been solved experimentally. However, several crystal structures of its homologs from other bacteria, e.g., *Thermus thermophilus* (PDB access code 2DQ4) and *Burkholderia thailandensis* (PDB accession code 5KIA), are available. The AlphaFold 3 model of *E. coli* TDH shows a homotetramer organized as a dimer of dimers, similar to other tetrameric bacterial alcohol dehydrogenases [21]. Each subunit consists of an N-terminal catalytic domain with a mixed α/β topology that is characteristic for alcohol dehydrogenases and a C-terminal cofactor-binding domain with a Rossmann fold. The dimer is formed from two subunits related by a two-fold axis (Figure 4a). In the tetramer, the two dimers are packed together face-to-face at an angle of about 90 degrees (Figure 4b). To connect the observed cooperative effects to the oligomeric structure of TDH, we analyzed a sample of TDH by mass photometry using the approximate concentrations used in the kinetic assays (10–40 nM final concentration). The enzyme population alone was partitioned between dimeric and tetrameric states (Figure 4c), with the estimated *K*_d_ value for the dimer-tetramer transition of 3 ± 1 nM. Because the dimeric form predominated at 10 nM concentration, we re-determined the kinetic parameters at a 10-fold higher enzyme (subunit) concentration (100 nM) to assess any changes in the cooperative binding of L-Thr due to a redistribution of the equilibrium between oligomeric forms in favour of the tetramer. Direct comparison of both saturation curves shows that they are essentially identical (Figure 4d). The determined kinetic parameters at 100 nM enzyme (subunit) concentration were *K*_0.5_ = 290 ± 10 µM, *k*_cat_ = 10.9 ± 0.1 s^−1^ and *h* = 1.4 ± 0.1 These results indicate that cooperativity is likely already present at the dimer level of TDH and that tetramer formation does not significantly affect its kinetic properties. Future experiments will be necessary for a detailed investigation of the molecular basis of cooperativity.

### 2.3. Inhibition of TDH by Compound ***1***

**Figure 5:**
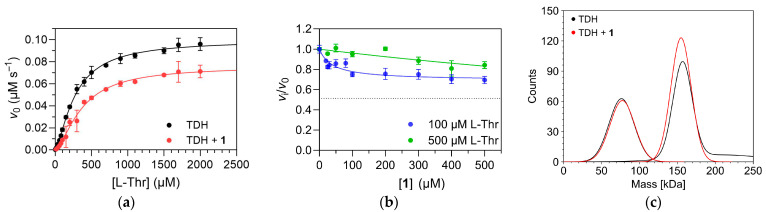
Effect of compound **1** on TDH activity. (**a**) Comparison of Michaelis–Menten plots in the absence and presence of compound **1** (100 µM final concentration) shows that both *K*_0.5_ and *V*_max_ are affected. (**b**) Titration of TDH with compound **1** in the presence of 100 µM L-Thr and 500 µM L-Thr, respectively, and 1 mM NAD^+^. All reactions were performed in 50 mM Tris-HCl buffer, pH 8.4, at 25 °C. The experimental points were fitted with the four-parameter logistic equation (Equation (2)). The dotted line represents the residual enzyme activity at saturation with compound **1** (*v*_inf_) at 100 µM L-Thr. At 500 µM L-Thr, this parameter could not be determined reliably due to the small overall changes in enzyme activity (~90% residual enzyme activity at 500 µM compound **1**). (**c**) Mass photometric analysis of the distribution of oligomeric states in the absence (black line) and presence (red line) of compound **1** (final concentration of 500 µM). The analysis was performed at a TDH subunit concentration of 20 nM in PBS buffer, pH 7.4, at 25 °C.

The effect of compound **1** on TDH activity was determined by comparing the activity of TDH as a function of L-Thr concentration in the presence or absence of compound **1** (Figure 5a) and by titrating TDH with compound **1** in the presence of fixed concentrations of L-Thr and NAD^+^ (Figure 5b). Two concentrations of L-Thr were selected for titration (100 µM and 500 µM, respectively) to determine the effect of increasing substrate concentration on inhibitory potency of compound **1**. Both concentrations are also comparable to the physiological concentration of L-Thr reported by Sander et al. which was in the 200–300 µM range [22]. The first set of experiments confirmed the inhibitory action of compound **1**. Both *K*_0.5_ and *V*_max_ were affected, indicating a mixed mechanism of action, or V-inhibition/K-inhibition according to Monod, Wyman and Changeux [23]. Similar kinetic mechanisms are characteristic, for example, of the well-known allosteric inhibition of phosphofructokinases from various organisms by metabolites [24]. The titration curve further showed that compound **1** is a partial inhibitor of TDH, with an *EC*_50_ value of 50 ± 10 µM at 100 µM L-Thr and 1 mM NAD^+^. The experimental points were fitted with the four-parameter logistic equation, which also contains the coefficient *h* to account for cooperativity effects and any deviations from a 1:1 binding stoichiometry between enzyme and inhibitor. However, the best-fit value of h was near 1, indicating that the binding of compound **1** to TDH is a non-cooperative process. The inhibitory activity of the compound was significantly diminished at 500 µM L-Thr, indicating a predominantly specific (competitive) mechanism of action. Altogether, compound **1** can thus be described as a partial mixed, predominantly specific inhibitor of *E. coli* TDH. The number and identity of the binding sites for compound **1** on TDH remain to be determined, but its kinetic mechanism of action indicates that it does not bind to the substrate binding site. Therefore, an allosteric mechanism of action is possible. We also used mass photometry to determine whether compound **1** affects the oligomeric equilibrium of TDH. No such effects were observed (Figure 5c), indicating that the binding site is also distant from the dimer-dimer interface.

## 3. Materials and Methods

### 3.1. Synthesis and Physico-Chemical Characterization of Compound ***1***

4-(2-aminoethyl)-2-(pyridin-2-yl)-1,2-dihydro-3*H*-pyrazol-3-one dihydrochloride (**1**) was prepared following a procedure from the literature [11]. Yield: 100%, white solid; mp 161–165 °C, lit. [12] mp 137–142 °C. ^1^H NMR (300 MHz, DMSO-d_6_): *δ* 2.60 (2H, br t, *J* = 7.3 Hz, 1′–CH_2_); 2.98 (2H, br sextet, *J* = 7.3 Hz, 2′–CH_2_); 7.30 (1H, ddd, *J* = 7.4, 5.0, 1.0 Hz, 5″–H); 7.72 (1H, s, 5–H); 7.99 (1H, ddd, *J* = 8.4, 7.4, 1.9 Hz, 4″–H); 8.13 (3H, br s, NH_3_^+^); 8.25 (1H, br d, *J* = 8.4 Hz, 3″–H); 8.45 (1H, ddd, *J* = 5.0, 1.8, 0.9 Hz, 6″–H); NH exchanged. Anal. Calcd for C_10_H_12_N_4_O·1.6HCl: C, 45.74; H, 5.22; N, 21.34. Found: C, 45.56; H, 4.98; N; 21.30; *m/z* (HRMS) Found: = 204.10111 (M^+^). C_10_H_12_N_4_O·requires *m/z* = 204.10185. ν_max_ (KBr) 3451, 3036, 2871, 1646 (C=O), 1628 (C=O), 1614, 1565, 1548, 1482, 1462, 1399, 1263, 1203, 1150, 934, 783 cm^−1^. Physical and spectral data are in agreement with data from the literature [12].

### 3.2. Dilution Antibiogram

The dilution antibiogram was used to determine the minimal inhibitory concentration for the antibacterial activity of compound **1** against *E. coli MG1655*. An overnight culture of *E. coli* was diluted 1000-fold into fresh LB broth and supplemented with increasing concentrations of compound **1**. Cultures were shaken for 5 h at 37 °C, and cell densities were then determined by measuring the optical density at 600 nm. The minimal inhibitory concentration was determined by visual inspection as the lowest concentration of compound **1** at which no bacterial growth was visible.

### 3.3. Disc Diffusion Assays

Disc diffusion assays were performed by the standard Kirby–Bauer method [22] on Mueller–Hinton agar plates (Merck, Germany) (90 mm diameter) with commercially obtained sterile 6 mm discs. Overnight cultures of *E. coli MG1655*, *Pseudomonas aeruginosa*, *Staphylococcus aureus RN1442,* and *Bacillus thuringiensis* were grown in LB medium, diluted to an OD_600_ of 0.1, and 100 μL each culture was evenly spread on a separate agar plate. Discs were placed evenly on the plates, and 3 μL of a 20 mg/mL solution of compound **1** in DMSO was applied to one disc. Positive and negative controls were applied on each plate, where the positive control was 3 μL of a 20 mg/mL solution of kanamycin, and the negative control was 3 μL of DMSO. Zones of inhibition were measured after overnight incubation of the plates at 37 °C.

### 3.4. Affinity Chromatography

Compound **1** was immobilized via its primary amino group on a 1 mL NHS-activated Sepharose 4 Fast Flow column (Cytiva, Marlborough, MA, USA) according to the manufacturer’s instructions. For immobilization, 10 mg of the compound were dissolved in coupling buffer (0.2 M NaHCO_3_, pH 8.3, containing 0.5 M NaCl) and passed through the column continuously for 3 h. For affinity chromatography, the column was equilibrated with 20 mM Na-phosphate buffer, pH 7.40, containing 0.5 M NaCl and 0.05% *v/v* Triton X-100. The sample was prepared by pelleting cells from a 400 mL overnight culture of *Escherichia coli* grown in LB medium at 37 °C, resuspending them in 30 mL of the buffer used for column equilibration, lysing the cells by sonication, centrifuging and finally filtering the clear supernatant through a 0.45 μm filter. The sample was applied to the column using a peristaltic pump at a flow rate of 1 mL/min. The column was then washed with 20 mL of equilibration buffer, and bound proteins were eluted with 10 mL of elution buffer (50 mM citric acid/Na-citrate, pH 3.0, containing 0.05% *v/v* Triton X-100). Fractions of 1 mL were collected during elution and analyzed by SDS-PAGE. Bound proteins were identified by trypsin digestion of proteins in the eluted fraction with the highest protein concentration, followed by LC/MS analysis of the fragments. The analysis was performed by a commercial service at the Jožef Stefan Institute (Ljubljana, Slovenia).

### 3.5. Cloning and Overexpression of Recombinant TDH

The TDH-coding sequence from *E. coli* strain DH5α was amplified by polymerase chain reaction (PCR) using the forward primer 5′-CCGGCTAGCATGAAAGCGTTATTCAAACTGAAAGC-3′ and the reverse primer 5′-CCGCTCGAGTTAATCCCAGCTCAGAATAAGTTTCCC-3′. The PCR product was ligated into the pJET 1.2 blunt vector (ThermoFisher Scientific, USA) via blunt ends, and correct amplification was verified by Sanger sequencing using a commercial service. The TDH coding sequence was then transferred to the pET-28b(+) expression vector (Merck Biosciences, Germany) using NheI and XhoI restriction sites included in the primers. Competent cells of *E. coli* strain BL21 (DE3) were transformed with the plasmid DNA. For protein expression, cells were grown in shaker flasks at 37 °C with shaking to an OD_600_ of 0.8, and expression was then induced with 0.3 mM IPTG. Induced cells were grown at 25 °C for 20 h and then harvested by centrifugation. Cells from 800 mL of culture were resuspended in 15 mL of binding buffer (20 mM Tris-HCl, pH 7.4, containing 500 mM NaCl and 20 mM imidazole), disrupted by sonication, and mixed with 1 mL of His Mag Sepharose Ni suspension (Cytiva, Marlborough, MA, USA). The magnetic beads were collected with a magnet and the unbound fraction discarded. The beads were washed with binding buffer and the bound proteins were finally eluted with 5 mL of elution buffer (20 mM Tris-HCl, pH 7.4, containing 500 mM NaCl and 500 mM imidazole). Eluted fractions were desalted and dialyzed on a disposable PD-10 desalting column (Cytiva, Marlborough, MA, USA) equilibrated in 50 mM Tris-HCl, pH 8.4, aliquoted, and stored at −80 °C. Protein concentration was determined from SDS-PAGE analysis and A_280_ measurements of the final samples.

### 3.6. Kinetic Measurements

Enzyme activity of TDH was followed fluorimetrically by measuring the fluorescence of NADH formed during the reaction between the substrate L-Thr and the co-factor/co-substrate NAD^+^ at an excitation wavelength of 340 nm and an emission wavelength of 440 nm. Reactions were performed in 50 mM Tris-HCl buffer, pH 8.4, at a final enzyme concentration of 10 nM in black 96-well microtiter plates. Fluorescence was measured using a BioTek Synergy H1M2 plate reader (Agilent, Santa Clara, CA, USA) in top-read mode at a fixed bandwidth of 9 nm. Progress curves were recorded for 3 min and were typically linear throughout the reaction. Reaction rates were therefore determined by linear regression. Plots of reaction rates versus substrate concentration were analyzed with the Michaelis–Menten equation modified to account for cooperativity:

(1) $$v_{0}=\frac{V_{max}{\left[S\right]}^{h}}{K_{0.5}^{h}+{\left[S\right]}^{h}}\;\;\;\;\;\;\;\;\;\;\;$$

where *v*_0_ is the initial reaction velocity, *V*_max_ equals $k_{cat}\cdot {\left[E\right]}_{t},\;$*h* is the Hill coefficient, and *K*_0.5_ is the substrate concentration at which $v_{0}=V_{max}/2$ and is equal to the Michaelis constant *K*_m_ when *h* = 1.

To characterize the effect of compound **1** on the activity of TDH, experiments were performed as above, except that activity was measured in the presence of varying concentrations of compound **1**, as indicated. The titration curves of TDH with compound **1** were fitted with the four-parameter logistic equation:

(2) $$v_{X}=v_{0}-\frac{\left(v_{0}-v_{inf}\right){\left[X\right]}^{h}}{{EC}_{50}^{h}+{\left[X\right]}^{h}}$$

where *v*_X_ and *v*_0_ are the initial reaction velocities in the presence and absence of compound **1**, *v*_inf_ is the theoretical initial reaction velocity at saturation with compound **1** (i.e., residual enzyme activity at saturation with compound **1**), *EC*_50_ is the concentration of compound **1** at which its effect is half-maximal, and *h* is the Hill coefficient. All calculations and visualizations of kinetic data were performed with GraphPad Prism 10 software (GraphPad Software, La Jolla, CA, USA).

### 3.7. Mass Photometry

Samples of TDH were analyzed at final protein concentrations between 10 nM and 40 nM in PBS buffer, with a pH of 7.4, at 25 °C in a Refeyn TwoMP mass photometer. Data was collected for 1 min and analyzed using the built-in software Discover^MP^ v2024 R2. The manufacturer’s MFP1 standard proteins were used for calibration. Calculation of equilibrium dissociation constants was performed as described in ref. [25].

### 3.8. Molecular Modelling

The three-dimensional molecular model of *E. coli* TDH was built using automated modelling with the AlphaFold 3 web server [26]. The protein sequence was retrieved from UniProt under accession number P07913. Visualization of the model was performed with UCSF Chimera X 1.10.1 software [27].

## 4. Conclusions

Antibiotic resistance is becoming a pressing concern in modern healthcare, especially as novel targets and types of antibiotics have been scarce in recent decades. Therefore, it is important to explore new ways of fighting bacterial infections and preventing bacterial growth in general. Herein we establish a link between the antibacterial activity of a 2-hydroxy-pyrazole compound and inhibition of TDH, which is involved in biofilm formation. This is especially promising since TDH does not have a functional human counterpart. The 2-hydroxy-pyrazole scaffold thus shows potential for further development of more potent broad-spectrum antibacterial agents. Moreover, we identify TDH as a cooperative enzyme and show that the compound is likely to act through an allosteric mechanism. These findings provide a promising starting point for further investigation of this under-investigated enzyme and the metabolic pathway(s) in which it is involved within cells.

## Figures and Tables

**Table 1:** Zones of inhibition (diameter in mm) measured for compound **1** against different bacterial strains. Kanamycin was used as the positive control and DMSO as the negative control.

	Compound 1	Kanamycin	Neg. Control
*Escherichia coli*	9.7 ± 0.9	21.7 ± 0.3	6
*Pseudomonas aeruginosa*	8.8 ± 0.2	8.3 ± 0.3	6
*Staphylococcus aureus*	8.2 ± 0.6	18.7 ± 0.9	6
*Bacillus thuringiensis*	8.2 ± 1.3	23.7 ± 0.7	6

**Table 2:** Kinetic parameters of recombinant *E. coli* TDH measured in 50 mM Tris pH 8.4 and 25 °C.

	*K*_m_/*K*_0.5_ (µM)	*V*_max_ (µM s^−1^)	*h*	est. *k*_cat_
L-Thr ^1^	280 ± 10	(10.0 ± 0.2) × 10^−2^	1.4 ± 0.1	10.0 ± 0.2
NAD^+ 2^	43 ± 3	(5.7 ± 0.1) × 10^−2^	1	5.7 ± 0.1

^1^ Determined at 1 mM NAD^+^ concentration; ^2^ determined at 1 mM L-Thr concentration.

## Data Availability

The original contributions presented in this study are included in the article. Further inquiries can be directed to the corresponding author.

## Abbreviations

The following abbreviation is used in this manuscript:

TDH	L-threonine 3-dehydrogenase
